# Mangiferin for the Management of Liver Diseases: A Review

**DOI:** 10.3390/foods12132469

**Published:** 2023-06-23

**Authors:** Lisi Li, Yujia Dong, Xifu Liu, Meng Wang

**Affiliations:** 1 Ministry of Education Key Laboratory of Molecular and Cellular Biology, Hebei Anti-Tumor Molecular Target Technology Innovation Center, College of Life Science, Hebei Normal University, Shijiazhuang 050024, China; lilisi95@163.com (L.L.); dyj131103@163.com (Y.D.); xfliu@hebtu.edu.cn (X.L.); 2 Key Laboratory of Ethnomedicine, Minzu University of China, Ministry of Education, Beijing 100086, China

**Keywords:** mangiferin, liver disease

## Abstract

The liver is a digestive and metabolic organ, and several factors can induce liver damage, which is a severe threat to human health. As a natural polyphenolic compound, mangiferin belongs to xanthone glucoside and mainly exists in many plants, such as mango. It is notorious that mangiferin has remarkable pharmacological activities such as anti-inflammatory, anti-tumor, antioxidative stress, antiviral and so on. Emerging evidence indicates the therapeutic benefits of mangiferin against liver disease, including liver injury, nonalcoholic fatty liver disease, alcoholic liver disease, liver fibrosis, and hepatocellular carcinoma. This review aims to summarize the possible underlying signaling mediated by mangiferin in liver disease treatment and the available findings of mangiferin, which can be used to treat different liver diseases and may contribute to mangiferin as a therapeutic agent for liver disease in humans.

## 1. Introduction

As a digestive and metabolic organ, the liver is involved in numerous physiological processes, such as nutrient metabolism, lipid homeostasis, and so on [1]. Several factors induce liver damage, including overnutrition, alcohol, and viral infection. Meanwhile, other organs may also lead to liver burdens, such as gut microbiota composition and adipose metabolism [2,3]. Liver disease is a public health concern that is responsible for over 2 million deaths and nearly accounts for 4% of all deaths worldwide [4]. The spectrum of liver disease ranges from steatosis to hepatitis and other advanced forms, including fibrosis, cirrhosis, and, eventually, liver cancer. Ectopic lipid accumulation in hepatocytes is the main characteristic of hepatic steatosis, which is thought to be the earliest response to damage factors. Hepatitis is usually defined as inflammation of the liver, which may be induced by alcohol, virus infections, and so on. More advanced damage factors activate hepatic stellate cells, which can lead to collagen deposition and liver scarring, causing the development of liver fibrosis and cirrhosis. In severe cases, liver cancer can develop, which is life-threatening [5]. Liver cancer is the most frequent fatal malignancy with poor prognosis, a high recurrence rate, and high mortality [6]. Therefore, fully understanding liver disease and finding safe and efficacious approaches are essential to block the onset and progression of liver disease.

Mangiferin (1,3,6,7-tetrahydroxyxanthone-C2-β-D-glucoside) is a natural polyphenolic compound that belongs to xanthone glucoside. It has been known that mangiferin is present in various plants, such as Anemarrhena and mangoes. Mangiferin is a bioactive compound with anti-inflammation, antioxidation, anti-diabetes, anti-tumor, anti-virus, antibacterial, anti-aging, and heart protection effects [7]. Notably, our study reported that mangiferin could ameliorate alcoholic fatty liver via the suppression of inflammation-induced adipose hyperlipolysis [8]. In addition, it has also been demonstrated that mangiferin could suppress liver fibrosis by inhibiting NF-κB signaling [9]. Mangiferin represses WT1-mediated LEF1 transcription to alleviate hepatocellular carcinoma [10]. Excluding mangiferin treatment alone, some combinations with mangiferin also exhibit positive effects in liver protection. Mangiferin, in combination with metformin and gliclazide, ameliorates hepatic enzyme expression and suppresses oxidative stress markers in HepG2 cells [11]. The complexation of mangiferin with soya phospholipid enhances hepatoprotection and the in vivo antioxidant activity in carbon tetrachloride-treated rats [12]. All these data indicate that mangiferin is a promising agent for the treatment of liver disease. Therefore, in the present review, we first listed the possible underlying signaling mediated by mangiferin in liver disease treatment. Additionally, we then summarized the available findings of mangiferin used in the treatment of different kinds of liver diseases, including liver injury, nonalcoholic fatty liver disease, alcoholic liver disease, liver fibrosis, and hepatocellular carcinoma.

## 2. Effect Targets of Mangiferin in Liver

### 2.1. NF-κB

It is widely accepted that inflammation responses play a crucial role in pathological processes, including liver damage. Nuclear factor-κB (NF-κB) is an essential member of inflammatory signaling. NF-κB is formed by the heterodimerization or homodimerization from the Rel family of DNA-binding proteins [13,14]. For example, the NF-κB heterodimer can be composed of p65 and p50, which regulates the transcription of genes containing κB binding sites [13]. However, the most crucial role of NF-κB is as a transcriptional regulator to regulate immunity and inflammation responses [13,14,15]. In the physiological state, the activity of NF-κB is mainly regulated by binding to the IκB proteins. The binding of IκB to NF-κB effectively sequesters NF-κB in the cytoplasm. Upon cellular stimulation, NF-κB can escape from the inhibitory protein IκB. Usually, the activation of Iκ-B kinase at serine residues (Ser32 and Ser36) leads to the liberation of NF-κB, allowing NF-κB to translocate into the nucleus [15]. Therefore, the phosphorylation and subsequent degradation of Iκ-B are essential for the nuclear translocation of NF-κB. Multiple factors can activate NF-κB, including lipopolysaccharide (LPS), carbon tetrachloride (CCl_4_), and inflammatory factors [15]. NF-κB activation is common in all chronic liver diseases, leading to severe hepatic pathology. It plays a pivotal role in both chronic liver disease and wound healing responses, which are essential for the proper functioning of the liver [15]. In addition, NF-κB in the liver can also activate hepatic stellate cells, thereby triggering the occurrence of liver fibrosis. Though NF-κB does not directly affect tumorigenesis, it plays a critical role in the prevention of hepatocytes’ transformation into hepatocellular carcinoma (HCC) [16]. These results provide compelling evidence that NF-κB is a promising target for different liver diseases. Notably, some natural plants and their metabolites exhibit inhibitory effects on NF-κB signaling [17]. Safranal, a major bioactive ingredient of saffron, can significantly inhibit NF-κB and other inflammatory cytokines, which has been shown to contribute toward protecting against diethylnitrosamine (DEN)-induced liver cancer in rats [18]. Previous studies have demonstrated that mangiferin exhibits excellent anti-inflammation capabilities in several diseases. Mangiferin inhibits IKKα/β kinase, leading to impaired IκB degradation and subsequently blocks the activation of the NF-κB classical pathway through translocation [19]. Mangiferin was found to suppress NF-κB, contributing to the protection of murine liver in Pb(II)-induced hepatic damage [20]. Mangiferin ameliorates acetaminophen-induced hepatotoxicity, which is also associated with NF-κB expression [21].

### 2.2. TGF-β/Smad

Smad proteins are direct receptors of the transforming growth factor (TGF)-β, which translocate from the cytoplasm to the nucleus to regulate the transcription of target genes [22]. Smad phosphorylation and activation in the cytoplasm are mediated by membrane serine/threonine kinase receptors in combination with TGF-β or related factors [23]. Upon phosphorylation, the Smad complex was transcribed into the nucleus, where it bound to specific DNA sequences in the target promoter and activated transcription. So far, now, there are three subgroups of Smad proteins that are known [23]. The first group is the receptor-regulated Smad proteins, including Smad2, Smad3, Smad1, Smad5, and Smad8. Smad2 and Smad3 can activate TGF-β, while Smad1, Smad5, and Smad8 are capable of phosphorylating bone morphogenetic protein (BMPs) receptors [24]. These proteins can be phosphorylated by the receptors and then associated with the second group, such as Smad4. Smad4 is a shared partner of receptor-regulated Smad proteins and is essential for TGF-β responses. Smad6 and Smad7 belong to the third group and act as decoys by binding to activated receptors and yielding inactive complexes. In this structure, Smad proteins are composed of two globular domains coupled by a linker region, including the amino-terminal domain and the carboxyl-terminal domain. The amino-terminal domain, or MH1 domain, is highly conserved in all receptor-regulated Smads and Smad4, except for Smad6 and Smad7. The carboxyl-terminal, or MH2 domain, is conserved in all Smad proteins, which are distinguished by junctions of variable length and sequences [25].

TGF-β, a member of the TGF-β family, is a crucial regulator of the cell cycle, differentiation, cell death, cell coding, and extracellular matrix associations. The TGF-β/Smad pathway has been demonstrated to be involved in various types of human cancers and mutations in TGF-β receptors or Smad proteins and can lead to the inactivation of the TGF-β/Smad pathway for the treatment of cancers [26]. For example, TGF-β signaling has been implicated in transforming from normal melanocytes into melanoma [27]. In addition, previous studies have demonstrated that the TGF-β/Smad pathway plays a crucial role in HBV-induced hepatocellular carcinoma [28,29]. HBV is an essential condition that leads to infection with cirrhosis and HCC: acute and chronic hepatitis [30]. The hepatitis virus infection induces TGF-β activation, which stimulates hepatic stellate cells producing a mass in the extracellular matrix and leading to fibrosis. It has been shown that ferulic acid alleviates liver fibrosis by regulating the TGF-β/Smad signaling pathway [31]. Ying Jiang et al. demonstrated that the up-regulation of hepatic Smad7 and downregulation of phosphorylation of Smad2 could inhibit the TGF-β/Smad signaling pathway, contributing to antifibrotic properties [32]. Mangiferin inhibited CCl_4_ and TGF-β1-induced liver fibrosis by blocking the TGF-β/Smad pathway to reduce cell epithelial-mesenchymal transitions [33]. Furthermore, mangiferin inhibits the WASP family verprolin-homologous protein (WAVE)-2 signaling pathway, promoting the inhibition of the hepatic stellate cell (HSC) by TGF-β1 suppression, thereby improving depressive behavior and tumor growth in colorectal liver metastases [34].

### 2.3. Nrf-2/Keap-1

It is well known that oxidative stress plays a pivotal role in the initiation and progression of many diseases, including liver disease [35]. There are two functions of molecular redox switches: one controlling the activation/deactivation of cycles and the other modulating or integrating with system activity. The nuclear factor-E2-related factor 2 (Nrf2) and Kelch-like ECH-associated protein 1 (Keap1) system belong to the redox-sensitive transcription factors and exhibit a broad range of biological functions. In mammals’ evolution, living organisms face the challenge of stressors, which induce functional defense systems’ formation. The Nrf2 and Keap1 systems inherited from ancestors are widely recognized as body defense systems that maintain cellular homeostasis [36]. Nrf2 is a homolog of nuclear factor-erythroid 2 p45, but the function of Nrf2 is not related to hematopoiesis. Nrf2 is a member of the cap-*n*-collar (CNC) basic leucine zipper protein and is a major sensor of oxidative stress in the cell [37]. Previous studies have found that oxidative stress response is significantly diminished in butylated hydroxyanisole-treated Nrf2 KO mice [38]. Keap1 is the main intracellular regulator of Nrf2. Keap1 is composed of five domains, including broad complex-tramtrack-bric and intervening region and glycine repeat domains, all of which are important for inhibiting Nrf2 activity [36]. Keap1 suppresses Nrf2 transcriptional activity by specific binding to its evolutionarily conserved amino-terminal regulatory domain [39]. Nrf2 constitutively accumulates and stimulates the transcription of target genes in the absence of Keap1 [40].

In a physiological state, Keap1 subjects Nrf2 to rapid ubiquitination and degradation, which inhibits Nrf2 transcriptional activity. However, under the stimulation of oxidative stress, the specific cysteinyl residues of Keap1 can be modified, leading to the loss of control of Nrf2. Nrf2 and Keap-1 can also decompose, and free Nrf2 can translocate to the nucleus, recognizing the antioxidant response element (AREs) and then activating downstream antioxidant genes, including heme Oxygenase-1(HO-1), NADPH-quinone oxidoreductase 1 (NQO1), and glutamate cysteine ligase (GCL) to protect against oxidative stress [41,42]. Importantly, some external factors can directly destroy or dissociate the Nrf2/Keap-1 complex, thereby hindering the antioxidant effect; therefore, the drugs that target the Nrf2/Keap-1 complex may be a potential treatment strategy for the oxidative stress-associated disease. It has been reported that the raspberry extract can protect against oxidative stress through the Nrf2-ARE-Keap-1 signaling pathway in HepG2 cells [43]. Notably, mangiferin is well known for its antioxidant effects. It has been demonstrated that mangiferin increases Nrf2 expression and nuclear translocation to protect against galactosamine-induced hepatic pathophysiology in rats [44]. Mangiferin also up-regulates the expression of Nrf2 and HO-1 in a dose-dependent manner in lipopolysaccharide and D-galactosamine-induced acute liver injury in mice [45].

### 2.4. NLRP3

Several studies have found the vital contribution of inflammasomes to the pathogenesis of various liver diseases [46,47]. Inflammasomes can recognize damaged cells—releasing the damage-associated molecular patterns (DAMPs)—and then induce the activation of caspase-1. Activating caspase-1 promotes the maturation of cytokines pro-IL-1β and pro-IL-18 to IL-1β and IL-18, which stimulates pyroptosis and the release of inflammatory factors into the extracellular space. The Leucine-rich repeat-containing proteins (NLR) family is one kind of inflammasome component that has been widely accepted to be involved in various experimental models and human liver diseases [46,48]. Among the different types of inflammasomes associated with liver disease, NLRP3-containing inflammasome has attracted more attention. The NLRP3 inflammasome is composed of three parts, including NLRP3, the apoptosis-associated speck-like protein containing a caspase-recruitment domain, and pro-caspase-1 [49]. A two-signal model has also been reported for NLRP3 inflammasome activation [50]. One occurs through the activation of the transcriptional factor NF-κB by endogenous cytokines or microbial molecules to upregulate NLRP3, and the other is provided by different stimuli, including toxins, adenosine triphosphate (ATP), viral RNA, and so on, all of which can activate the NLRP3 inflammasome.

Multiple studies have investigated the NLRP3 inflammasome in hepatic inflammation and hepatocyte damage [51]. Paul Kubes et al. reported that sterile inflammation is a key process in various liver diseases, including drug-induced liver injury, alcoholic steatohepatitis, and nonalcoholic steatohepatitis, and it is also a major determinant in liver fibrosis and carcinogenesis [52]. Alexander Wree et al. assessed the effects of persistent NLRP3 activation as a contributor to NAFLD development and discovered that NLRP3 knockout mice exhibited significant protection from choline-deficient amino acid-defined diet-induced liver fibrosis, and they also found that patients with severe NAFLD exhibit increased levels of NLRP3 inflammasome components, all of which indicates the crucial role of the NLRP3 inflammasome in the development of NAFLD [53]. However, a previous study reported that mangiferin suppressed the activation of the NLRP3 inflammasome and restrained the expression of NLRP3 inflammasome-related proteins, which was implicated in inflammation effects and cell pyroptosis, contributing to the alleviation of high-fat diet-induced NAFLD in mice [54]. Chenwei Pan et al. found that mangiferin protected against lipopolysaccharide and D-galactosamine-induced acute liver injury by activating the Nrf2 pathway and inhibiting NLRP3 inflammasome activation [45]. Mengran Li et al. measured NLRP3 gene expressions in mangiferin-treated alcohol hepatitis rats and found that mangiferin down-regulated NLRP3 gene expression compared to the alcohol group [55]. All these data demonstrate the positive effects of mangiferin on the liver disease through NLRP3 signaling.

### 2.5. AMPK

Amp-activated protein kinase (AMPK) is a key enzyme in regulating glucose and lipid metabolism [56], and it is involved in systemic energy metabolism. AMPK is a heterotrimer that is composed of three subunits: an α-subunit, which phosphorylates the NH2-terminus (Thr172); a β-subunit, which is present at the COOH-terminus and acts as the major scaffold subunit that maintains AMPK; and a γ-subunit, which regulates the allosteric, ATP-inhibitory, AMP-and ADP-activated kinases, which is also known as regulatory subunits of AMPK [57,58,59]. The liver is an important organ for lipid metabolism, while the AMPK signaling pathway plays a pivotal role in lipid metabolism regulation in the liver [60]. AMPK affects lipid metabolism mainly by inhibiting the activities of malonyl-CoA and acetyl-CoA through fatty acid oxidation and reducing triglyceride synthesis. Additionally, AMPK monitors intracellular ATP levels and accelerates the production of ATP when facing reduced ATP levels. Energy change is an important factor for the occurrence of diseases; therefore, AMPK is a potential biological target that plays a therapeutic role in metabolism disease. AMPK has been shown to improve the autophagy ability of hepatocytes by up-regulating the expression of the ubiquinol-cytochrome c reductase core protein 2 (UQCRC2) protein to alleviate alcoholic liver disease [61], indicating AMPK to be an important target for the treatment of liver diseases. AMPK has also been demonstrated to reduce the symptoms of nonalcoholic fatty liver by reducing hepatic gluconeogenesis and increasing glucose uptake in the muscle tissue [62]. The major upstream kinase that regulates the AMPK activity in the liver is serine/threonine kinase liver kinase B1(LKB1): a known tumor suppressor [63]. Additionally, AMPK activity is associated with inflammation, which is vital for preventing and treating nonalcoholic fatty liver disease [62]. Furthermore, AMPK is known to phosphorylate sterol regulatory element-binding proteins (SREBP-1c and -2), providing new methods to combat hepatic steatosis and atherosclerosis in diet-induced insulin-resistant mice [64]. In line with this, the mango tree leaf extract regulates lipid and glucose homeostasis by AMPK and PI3K/AKT signaling pathways [65]. Yi Zhang et al. found that mangiferin activates sirtuin-1 and liver kinase B1 along with increasing the AMP/ATP ratio intracellularly, followed by AMPK phosphorylation, leading to decreased triglyceride content [66]. Previous studies have demonstrated that mangiferin alleviates plasma FFA levels by promoting free fatty acid (FFA) uptake and oxidation by regulating hepatic AMPK pathway signaling. Based on these investigations, we found AMPK to be a major target of mangiferin to improve hepatic metabolism, which contributed to the treatment of liver disease.

### 2.6. Other Targets

In addition to the targets mentioned above, there are a variety of other potential mechanisms contributing to mangiferin that can protect against liver diseases. Jihyeon Lim et al. reported that mangiferin increased proteins expression with mitochondrial biogenesis and oxidative activity, including oxoglutarate dehydrogenase E1 (Dhtkd1) and the cytochrome c oxidase subunit 6B1 (Cox6b1). Meanwhile, mangiferin also down-regulated the protein expression associated with lipogenesis, including fatty acid stearoyl-CoA desaturase 1 (Scd1) and acetyl-CoA carboxylase 1 (Acac1) [67]. Mangiferin was demonstrated to ameliorate arsenic-induced apoptosis in the liver by altering the Bax–Bcl-2 ratio and suppressing the mitochondrial pathway in relation to apoptotic proteins [68]. Moreover, mangiferin has been shown to inhibit mitogen-activated protein kinases (MAPKs), including phospho-ERK 1/2, phosphor-JNK, and phospho- p38 to protect the murine liver in Pb(II)-induced liver damage [20]. Mangiferin can effectively regulate the expression levels of specific alcohol hepatitis-associated genes, potential biomarkers, and metabolic pathways in alcohol hepatitis rats [55]. Lastly, PPAP-γ is a cell differentiation transcription factor with multiple functions, such as regulating glucose and lipid metabolism, anti-inflammation, and reducing oxidative stress. PPAP-γ has been shown to interact with NF-κB, thereby modulating the balance of M1/M2 macrophages and helping to slow the progression of various inflammatory-associated diseases [69]. It was reported that mangiferin mediated the Wnt/β-catenin/NF-κβ/PPAR-γ signaling pathway, which was involved in regulating oxidative stress, inflammation, and apoptosis to protect against intestinal ischemia/reperfusion-induced liver injuries in rats [70]. 

## 3. Mangiferin Protects against Liver Disease

### 3.1. Liver Injury

One important function of the liver is to metabolize the drugs and toxins. It is widely recognized that the liver is the most frequently targeted organ for drugs and toxins and faces a toxic threat directly [71]. The familiar exogenous toxins include organic solvents and drugs. Acute liver injury refers to the rapid abnormality of hepatocytes under the stimulation of some external factors, such as organic solvents and drug abuse [72]. Additionally, long-term liver injuries can lead to the development of chronic hepatitis and liver fibrosis. So far, now, drug-induced liver damage remains a challenge in clinical practice, and more than 1000 drugs are known to have side effects on the liver [73]. Excluding chemical toxin-caused liver damage, organic solvents can induce liver damage by releasing free radical species such as oxygen species: a potential indicator of hepatotoxicity [74]. The excess production of reactive oxygen triggers the imbalance of redox balance in hepatocytes, leading to oxidative stress. Oxidative stress is thought to be the pathophysiology of chronic liver disease etiologies, contributing to the development of liver injury progression [75]. The inflammatory response also plays an important role in chemical toxicities that pose a challenge in the liver and promotes the progression of liver disease by activating Kupffer cells. Activated Kupffer cells produce large amounts of inflammatory cytokines, such as tumor necrosis factor (TNF)-α, interleukin (IL)-1, and IL-6 [76].

Among the chemical toxicity factors, carbon tetrachloride-(CCl4-) induced hepatotoxicity is the most common model in studies [77]. CCl_4_ is activated by the cytochrome P450 enzyme (CYP2E1) in the endoplasmic reticulum (ER) to form the trichloromethyl radical (CCl3•). CCl3• is highly reactive and can react with oxygen, resulting in lipid peroxidation and hepatic injury [78]. Previous studies reported that oxidative stress and inflammation response occurred in CCl_4_-induced liver injury [79]. The CCl_4_ challenge was downregulated with antioxidant enzymes, including glutathione (GSH), glutathione-S-transferase (GST), catalase (CAT), and superoxide dismutase (SOD), along with elevated inflammation cytokines, including TNF-α, IL-1β and IL-6 in mice [79]. Interestingly, several studies have demonstrated the effects of mangiferin on oxidative stress and inflammation [80]. It has been reported that mangiferin alleviated acute liver injury by promoting the antioxidant HO-1 and reducing the production of TNF-α [81,82]. All these indicate the positive effect of mangiferin on protecting against liver injury.

### 3.2. Nonalcoholic Fatty Liver Disease

Nonalcoholic fatty liver disease (NAFLD) is the most common liver disease worldwide, with an estimated prevalence of 25% [83]. Several risk factors can induce NAFLD, including drugs, viral infections, and autoimmunity [84]. NAFLD can be classified into nonalcoholic fatty liver, nonalcoholic steatohepatitis, and more severe pathological changes. The main character or the initial pathological progression is lipid accumulation in the liver. The excessive accumulation of lipids leads to the excessive accumulation of toxic substances in lipid metabolism, which further blocks the mitochondrial respiratory chain electron flow, forms reactive oxygen species (ROS), and promotes liver damage [75,85]. Except for alcohol, other risk factors that cause lipid accumulation in the liver are when energy intake is not balanced with energy expenditure. Patients with nonalcoholic fatty liver usually exhibit liver steatosis and mild nonspecific inflammation together. Therefore, nonalcoholic fatty liver may develop into nonalcoholic steatohepatitis and even cirrhosis or liver cancer without drug intervention [24]. Nonalcoholic steatohepatitis is one of the most progressive subtypes of NAFLD. As metabolic-associated fatty liver diseases, nonalcoholic steatohepatitis is characterized by steatosis, which is linked to metabolic syndrome, diabetes, obesity, and dyslipidemia-mediated disorders [86].

It is well known that NAFLD is a multisystem disease [87]. Excess lipid accumulation in the liver impairs insulin resistance, leading to hepatic metabolic abnormalities and increasing the risk of type 2 diabetes [88,89,90,91]. In addition, NALFD can also affect cardiovascular and cerebrovascular diseases and kidney diseases [87,92,93,94,95]. The factors that relate to metabolic syndromes, such as obesity, diabetes, and hyperlipidemia, can lead to lipid ectopic accumulation and to a fatty liver [96]. Mangiferin plays an important role in preventing hyperglycemia and obesity induced by a high-fat diet in mice [97,98,99,100]. Jihyeon Lim et al. reported that mangiferin prevented HFD-induced liver steatosis and adiposity by increasing mitochondrial bioenergetics and oxidative activity-associated proteins oxidative pentylene glycol dehydrogenase (Dhtkd1) and cytochrome c oxidase subunit 6B1 (Cox6b1). Meanwhile, mangiferin downregulated proteins controlling de novo lipogenesis fatty acid stearoyl-coa desaturase 1(Scd1) and acetyl-coa carboxylase (Acac1) [67]. In line with this, Zhang Yong et al. found that mangiferin alleviated NAFLD in mice by regulating glucolipid metabolism by AMPK activation and suppressing the inflammation response of NLRP3 inflammasome inhibition [54]. These studies suggest that mangiferin is a promising prodrug for the treatment of NAFLD patients.

### 3.3. Alcoholic Liver Disease

Alcoholic liver disease (ALD) is one of the leading causes of chronic liver disease worldwide, which is mainly caused by long-term excessive alcohol consumption [101]. Alcohol disrupts normal liver functions and leads to hepatic structural damage, eventually resulting in ALD. As a vital digestive organ, the liver is the primary organ that is exposed to ethanol absorption and metabolism. Though alcohol can be metabolized by alcohol-metabolizing enzymes in the gastrointestinal tract, over 80% of abused alcohol is oxidatively metabolized in the liver. There are more alcohol metabolizing enzymes, including alcohol dehydrogenase, CYP2E1, and catalase, which are expressed in hepatocytes. In hepatocytes, ethanol was first oxidized to acetaldehyde. The conversion from ethanol to acetaldehyde is relatively short-lived and is subsequently metabolized to acetate. It is worth noting that acetaldehyde is more toxic than ethanol. Acetaldehyde covalently binds to biomacromolecules such as proteins, nucleic acids, and phospholipids to form adduct formations, altering protein functions [102]. It is important to note that alcohol metabolizing enzymes catalyze the oxidation of alcohol, leading to the reduction of NAD+ and the forming of nicotinamide adenine dinucleotide (NADH). The lower ratio of intrahepatocyte NAD+/NADH, which is also called cellular redox potential, alters the metabolic shift from the metabolism toward fatty acid synthesis in hepatocytes. Chronic drinking elevates the fatty acid contents, which form lipid droplets in the liver, resulting in alcoholic hepatic steatosis. So far, as of now, there is no Food and Drug Administration (FDA)-approved drugs used for the treatment of alcoholic liver disease, and the main strategy is the control of alcohol intake. As the initial stage of ALD and a reversible pathological condition, alcoholic hepatic steatosis treatment attracts more and more attention. Our previous study investigated the effects of mangiferin on ethanol-induced liver injury using a chronic plus binge ethanol mouse model. We found mangiferin in adipose through ameliorating phosphodiesterase 3B (PDE3B) stability by AMPK/ noncanonical NF-κB signaling, which contributed to the prevention of alcoholic liver disease [8]. In addition, Mengran Li et al. reported mangiferin ameliorates hepatic damage-associated molecular patterns, a lipid metabolic disorder, and mitochondrial dysfunction in alcohol hepatitis rats by regulating specific alcohol hepatitis-related genes, potential biomarkers, and metabolic pathways [55].

### 3.4. Liver Fibrosis

Liver fibrosis is an advanced pathological process following chronic liver injury. As a serious health problem, liver fibrosis mainly determines the quality of life, which may result in advanced liver cirrhosis and hepatocellular carcinoma. The main characteristic of liver fibrosis is a progressive accumulation in the extracellular matrix and the accumulation of the extracellular matrix, which destroys the hepatic physiological function. Liver fibrosis is caused by different factors such as alcohol, virus, cholestasis, and so on. These risk factors stimulate chronic inflammation, which induces an abnormal wound-healing response and forms fibrous scars [103,104]. Myofibroblast activation and proliferation is the main reason for fibrogenesis because myofibroblasts are responsible for extracellular matrix production in the damaged liver. It is worth noting that hepatic stellate cells are a major source of myofibroblasts though these are not unique precursors. In the physiological state, hepatic stellate cells keep quiescent, and their major function is to store vitamin A in the liver. As a result of liver injury, chronic inflammation may activate hepatic stellate cells, leading to fibrogenesis. Excluding the inflammation cytokines, ROS and acetaldehyde can also directly activate HSCs and stimulate immune cells to promote fibrotic mediators, leading to fibrosis [105,106,107]. Chronic hepatocyte injury can also lead to the release of damage-associated patterns (DAMPs) and apoptotic bodies, thereby activating hepatic stellate cells [5]. Risk factors can also recruit immune cells and activate Kupffer cells, which have a complex interaction with each other and promotes myofibroblast differentiation to induce extracellular matrix production [108]. Xiaoling Zhang et al. found that mangiferin could inhibit CCl_4_ and TGF-β1-induced liver fibrosis through decreasing heat shock protein (HSP)27 expressions to suppress the janus kinase/signal transducer and activator of the transcription (JAK2/STAT3) pathway, which downregulated TGF-β1/Smad signaling and then contributed to liver protection [33]. Similarly, Lijun Zhang et al. reported that mangiferin alleviated collagen accumulation and HSCs activation in the liver of CCl_4_-challenged mice by inhibiting NF-κB signaling [9].

### 3.5. Hepatocellular Carcinoma

HCC is the most common fatal malignant tumor worldwide, and it is ranked as one of the six most common cancers in the world due to its poor prognosis and high mortality [6]. HCC is a primary malignant cancer of liver cells. In the early stage of HCC, surgical resection, chemotherapy, and liver transplantation can be used to intervene. At the same time, tyrosinase inhibitors are the only treatment for advanced HCC; however, the prognosis is abysmal [109]. HCC often occurs in the background of chronic liver diseases and cirrhosis. There are many causes of HCC, among which hepatitis B virus infection and hepatitis C virus infection are the most important causes, accounting for 80% of the global population [110,111]. In developed countries, NAFLD is one of the most dangerous factors leading to HCC [112]. While in Europe and America, alcohol, as a common external factor that is harmful to the liver, is the second most common cause of liver cancer [113]. In addition, aflatoxin and aristolochic acid, which have strong carcinogenic effects, can also lead to HCC [111,114]. Mangiferin exhibits excellent capability for the treatment of hepatocellular carcinoma. It has been reported that mangiferin suppresses orthotopic tumor growth and HCC expansion and invasion through the β-catenin-independent Wnt signaling pathway to regulate related proteins in hepatocellular carcinoma cells [10]. Guang Yang et al. investigated the anticarcinogenic property of mangiferin against diethylnitrosamine-induced hepatocellular carcinoma in Sprague Dawley rats [115]. They found that mangiferin modulated lipid peroxidation levels and increased the endogenous antioxidant defense mechanisms in diethylnitrosamine-stimulated hepatocellular carcinogenesis. They also found mangiferin could markedly induce apoptosis and alleviate tumor markers levels in Sprague Dawley rats.

## 4. Conclusions

**Figure 1:**
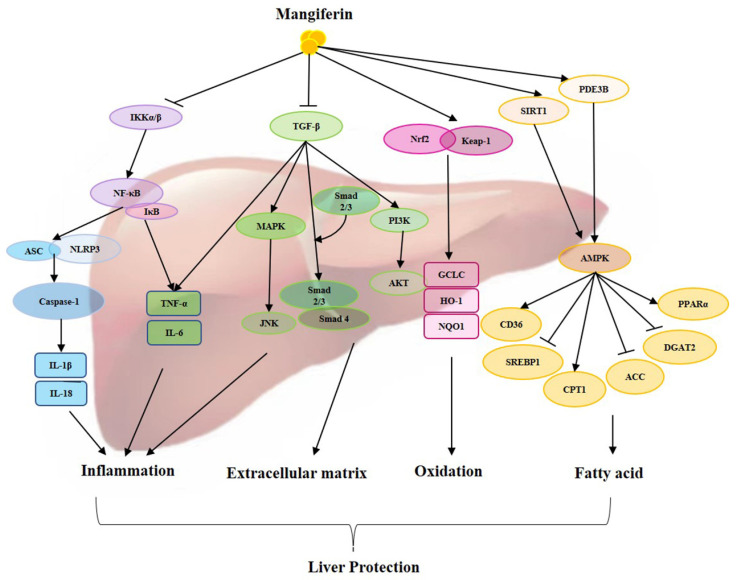
Overview of protective actions of mangiferin against liver diseases through different signaling pathways.

As a traditional medicine, mangiferin exhibits a wide range of biological activities and attracts more and more attention for the treatment of diseases, including liver diseases. The possible mechanism of mangiferin on hepatic protection has been extensively studied, mainly focusing on the capability of antioxidants, anti-inflammatories, suppressing the extracellular matrix, regulating fatty acid metabolism, and other specific signaling pathways (Figure 1). This review describes the different pharmacological activities of mangiferin in the treatment of liver diseases by affecting different mechanisms, including NF-κB, TGF-β/Smad, Nrf-2/Keap-1, NLRP3, AMPK, and others. Mangiferin has exhibited protective effects on liver injury, nonalcoholic fatty liver disease, alcoholic liver disease, liver fibrosis, and liver cancer (Table 1). All these indicate that mangiferin has a vital regulatory function in the liver and plays an important role in treating liver diseases.

Mangiferin significantly reduced liver damage as well as inflammation, extracellular matrix, oxidation, and fatty acid pathways. Abbreviations: ACC—acetyl-CoA carboxylase; AKT(PKB)—protein Kinase B; AMPK—AMP-activated protein kinase; ASC—apoptosis-associated speck-like protein containing a CARD; CPT1—carnitine palmitoyltransferase 1; CD36—fatty acid translocase; DGAT2—diacylgycerol acyltransferase 2; GCLC—glutamate cysteine ligase; HO-1—heme oxygenase-1; IKKα/β—IκB kinase α/β; IκB—inhibitor kappa B; IL-1β—interleukin-1β; IL-18—interleukin 18; IL-6—interleukin 6; JNK—jun N-terminal kinase; Keap-1—kelch-like ECH—associated protein 1; MAPK—mitogen-activated protein kinases; NF-κB—nuclear factor kappa-B; Nrf2—nuclear factor erythroid 2-related factor; NLRP3—nucleotide binding oligomerization domain associated protein 3; NQO1—NADPH-quinone oxidoreductase 1; PDE3B—phosphodiesterase 3B; PI3K—phosphoinositide 3 kinase; PPARα—peroxisome proliferator-activated receptor-alpha; SREBP1—sterol regulatory element-binding proteins; SIRT1—sirtuin 1; TGF-β—transforming growth factor; TNF-α—tumor necrosis factor.

## Figures and Tables

**Table 1:** A summary of mangiferin in liver disease. (“↓” and “↑” indicate significantly decrease and increase, respectively (*p* < 0.05)).

Compounds	Model	Cell Lines/Animals	Mechanism	Reference
Mangiferin	LPS and D-GalN-induced acute liver injury	BALB/c, Primary hepatocytes	Nrf2↑ NLRP3↓ ASC↓ Caspase-1↓ IL-1β↓ HO-1↑ TNF-α↓ MCP-1↓ ROS↓	[45]
Mangiferin	Against arsenic (As)-induced oxidative damages in the murine liver	Wistar rats, HepG2 cells	ROS↓ Caspase↓ AKT, Nrf2↑ PI3K↓ ERK1/2↓	[68]
Mangiferin	Mesenteric ischemia/reperfusion (I/R)-induced liver injury	Wistar rats	β-catenin↑ PPAR-γ↑ GSK-3β↓ NF-κB-p65↓ IL-6↓ Bcl-2↑ IL-1β↓ Caspase-3↓	[70]
Mangiferin	LPS/D-GalN-induced acute liver injury mouse model	BALB/c	HO-1↑ TNF-α↓ TLR4/NF-κB↓	[78]
Mangiferin	CCl_4_-injured liver model, BDL	C57BL/6J, knockdown of Nrf2 C57BL/6J, Primary hepatocytes	Nrf2↑ HO-1↑ NQO1↑ TNF-α↓ IL-1β↓ IL-6↓ ROS↓ mtDNA↓ NF-κB↓ TLR9/MyD88↓	[82]
Mangiferin	LPS/D-GalN-induced acute liver injury mouse model	BALB/c, Primary hepatocytes	miR-20a↓ miR-101a↓ Nrf2↑	[80]
Mangiferin	High-fat diet (HFD) (NAFLD)	C57BL/6J, HepG2 cells	AMPK↑ NLRP3↓ Caspase-1↓ IL-1β↓	[54]
Mangiferin	HFD (NAFLD)	Kunming mice	NF-κB↓ p-JNK↓ AMPK/mTOR↑ IRS/PI3K/AKT↑	[99]
Mangiferin	HFD (hyperlipidemia)	C57BL6/J	Dhtkd1↑ Cox6b1↑ Scd1↓ Acac1↓	[67]
Mangiferin	STZ-nicotinamide-NA-induced diabetic rats (Type 2 diabetes)	Albino Wistar rats	HbA1c↓ PPAR-γ↑ FALDH↑	[88]
Mangiferin-berberine (MB) salt	OA induced HepG2 cells	HepG2 cells	p-AMPK𝛼 (Thr172)/p-ACC (Ser79) ↑ CPT1↑	[95]
Isolated from the leaves of Salacia oblonga	STZ-induced diabetes rat	Albino rats	PPAR-γ/GLUT4↑	[89]
Mangiferin	HFD-Fr-STZ (type 2 diabetes)	Wistar rats	TNF-α↓ PPAR-α↑ SREBP-1↓ AMPK↑ MTP↓ GSK3β↑	[90]
Mangiferin	T2DM model rats, IR model of HepG2 cells	SD rats, HepG2 cells	PPAR-γ↑ GLUT4↑ AMPK↑ TNF-α↓ NF-κB↓ NLRP3↓ COX2↓ MAPK IL-6↓ IL-1β↓ NO↓ PI3K/AKT↑	[91]
Mangiferin	HFD, oleic acid (lipid metabolism)	Wistar rats, HepG2 cells	DGAT↓ CPT1↑ CD36↑ ACC↓ AMPK↑	[92]
Mangiferin	HFD (obesity and glucose metabolism)	C57BL/6	F4/80↓ TNF-α↓ FGF21↑ ATG7↑ JNK↑ IL-6	[93]
Mangiferin	HFD (hyperlipidemia)	hamsters	CPT1↑ PPAR-α↑ CD36↑ ACC↓ SREBP-1↓ MTP↓ DGAT2↓	[94]
X-3, a derivative of mangiferin,	db/db (C57BL/KsJ), db/ (+) (C57BL/KsJ) mice,	C57BL/6J, Murine 3T3-L1 preadipocytes, LKB1-deficient Hela cells, and HEK293 cells	AMPK↑ AKT↑ ACC↑	[97]
Mangiferin	HFD (liver inflammation caused by fat-rich diets)	Wistar rats	PPAR-α↑ HSP27↑ p65↓ IκB↓ TNF-α↓ IL-1β↓ iNOS↓ IL-10↓	[98]
Mangiferin	Gavaged with a single dose of ethanol	C57BL/6	PDE3B↑ NF-κB↓ AMPK/TBK1↑ CD36↓ FATP2↓ FATP5↓ ATGL↓ SREBP-1↓ p65↓ p100↓ ULK1↑	[8]
Mangiferin	Alcohol hepatitis (AH) rats	Sprague–Dawley rats	PPAR-γ↑ OPA-1↑ Cav-1↑ EB1↓ NF-κB↓ p65↓ NLRP3↓ Cas-1↓ IL-1β↓ HSP90↓ HMGB1↓ SYK↓ CCL20↓ C-CAS-3↓ C-PARP↓ STARD1↓	[55]
Mangiferin	CCl_4_-injured liver model	C57BL/6J	IL-6 IL-1β↓ α-SMA↓ TGF-β↓ MMP-2↓ ABCB4↓ ABCB11↓ SULT2A1↓ p-IκB↓ p-p65↓ NF-κB↓	[9]
Mangiferin	CCl_4_-injured liver model	rats	HSP27↓ JAK/STAT↓ Smad/TGF-β↓	[33]
Mangiferin	D-GAL induced hepatic pathophysiology	Swiss albino male rats	TNF-α↓ IFN-γ↓ IL-1β↓ IL-6↓ IL-12↓ IL-18↓ IL-10↓ iNOS↓ NF-κB↓	[44]
Mangiferin	Orthotopic HCC implantation murine model,	MHCC97L Cells, BALB/c-nu/nu,	WTI↓ LEF1↓ MYC↓ Axcin2↓ MMP2↓ CCND1↓	[10]
Mangiferin	DEN-induced hepatocellular carcinoma	Sprague Dawley rats	ROS↓ Bcl-2↓ Bax↑ Caspase-3↑ 8-OHdG↓ NF-κB	[115]
Mangiferin	APAP-induced hepatotoxicity	C57BL/6	TNF-α↓ IL-6↓ MCP-1↓ CXCL-1↓ CXCL-2↓ IL-1β↓ NF-κB↓ AMPK↑	[21]
Mangiferin	Galactosamine-induced hepatic toxicity	SHRs/NCrlVr	DGAT↓ CPT1↑ PPAR-α↑ CD36↑ MTTP↓	[116]

## Data Availability

No new data were created or analyzed in this study. Data sharing is not applicable to this article.

## Abbreviations

acetyl-CoA carboxylase 1 (Acac1), alcoholic liver disease (ALD), amp-activated protein kinase (AMPK), acetaminophen (APAP), antioxidant response element (AREs), adenosine triphosphate (ATP), bile duct ligation (BDL), bone morphogenetic proteins (BMPs), catalase (CAT), trichloromethyl radical (CCl_3_•), carbon tetrachloride (CCl_4_), cap-n-collar (CNC), cytochrome c oxidase subunit 6B1 (Cox6b1), cytochrome P450 enzyme (CYP2E1), damage-associated molecular patterns (DAMPs), diethylnitrosamine (DEN), deoxyribonuclease 2 (DNase 2), oxoglutarate dehydrogenase E1 (Dhtkd1), endoplasmic reticulum (ER), food and drug administration (FDA), glutamate cysteine ligase (GCL), hepatitis B virus (HBV), hepatocellular carcinoma (HCC), heme oxygenase-1 (HO-1), hepatic stellate cell (HSC), heat shock proteins 27 (HSP27), high-fat diet (HFD), interleukin (IL)-1, janus kinase/signal transducer and activator of transcription (JAK2/STAT3), kelch-like ECH-associated protein 1 (Keap1), liver kinase B1(LKB1), lipopolysaccharide (LPS), mitochondrial DNA (mtDNA), mitogen-activated protein kinases (MAPKs), myeloid differentiation factor88 (MyD88), nicotinamide adenine dinucleotide (NADH), nonalcoholic fatty liver disease (NAFLD), nuclear factor kappa-B (NF-κB), nucleotide- binding oligomerization domain associated protein 3 (NLRP3), NADPH-quinone oxidoreductase 1 (NQO1), nuclear factor erythroid 2-related factor (Nrf2), phosphodiesterase 3B (PDE3B), stearoyl-CoA desaturase 1 (Scd1), streptozotocin- (STZ-), superoxide dismutase (SOD), reactive oxygen species (ROS), sterol regulatory element-binding proteins (SREBP-1c and -2), transforming growth factor (TGF)-β, Toll-like receptor-9 (TLR9), tumor necrosis factor (TNF)-α, ubiquinol-cytochrome c reductase core protein 2 (UQCRC2), WASP family verprolin-homologous protein (WAVE).
